# Prof. Dr. Giovanni De Toni—Editorial for the Commemoration of a Pediatric Luminary

**DOI:** 10.3390/ijms26073198

**Published:** 2025-03-30

**Authors:** Consolato M. Sergi, Angelo Ravelli, Luca Pio

**Affiliations:** 1 Division of Anatomic Pathology, Children’s Hospital of Eastern Ontario, University of Ottawa, Ottawa, ON K1N 6N5, Canada; 2 Department of Laboratory Medicine and Pathology, University of Alberta, Edmonton, AB T6G 2R3, Canada; 3 Scientific Direction, Institute G. Gaslini, 16147 Genova, Italy; angeloravelli@gaslini.org; 4 Department of Surgery, St. Jude Children’s Research Hospital, 262 Danny Thomas Place, Memphis, TN 38105, USA; luca.pio@stjude.org

Living in Liguria, acknowledging that the sea may be a metaphor for the human condition is not difficult. The 1975 Nobel Prize winner for literature, Eugenio Montale, was born in Genoa, Italy, in 1896, and spent his childhood between his hometown and the picturesque village of Monterosso, Cinque Terre. Paraphrasing Mengaldo, the ego feels almost pulled in by the sea, and simultaneously, it is ejected and confined to the land. Montale’s collection “Ossi di Seppia” is an evocation and allegory of the misery and marginalization of the human condition which moved Giovanni De Toni to action. Two years before Montale won his Nobel Prize for literature, Giovanni de Toni, an outstanding pediatrician and director of one of the largest children’s hospitals worldwide, passed away. Born in Venice in 1895, Giovanni De Toni was an Italian pediatrician. Known internationally for describing De Toni–Fanconi–Debré syndrome, he was a prominent figure in Italy, so much so that the Department of Pediatric Sciences of the University of Genoa bears his name. With his wife, Prof. Dr. De Toni also launched the “Villa Santa Chiara” in Genoa to assist disabled children. In 1933, he described a new type of pathology, later known as “De Toni–Fanconi–Debré syndrome”, in which the bone-derived hormone fibroblast growth factor-23 (FGF23) inhibits the kidney’s ability to reabsorb phosphate and produce vitamin D [1,2,3,4]. A basic impairment in the function of the renal proximal tubule cells causes hyperphosphaturia, renal tubular acidosis, glycosuria, and widespread aminoaciduria in primary De Toni–Fanconi–Debré syndrome, a non-FGF23-mediated hypophosphatemic condition [5,6,7,8,9,10,11,12].

Later, he characterized children affected by hyperosteogenesis, and was an authentic innovator in auxologic studies. According to many scholars, Professor De Toni identified a form of infantile cortical hyperostosis, also studied in 1945 by John Caffey and William Silverman (so-called “Caffey–De Toni disease”). In 1942, Prof. De Toni became the director at the “Istituto G. Gaslini” in Genoa, where he creatively transformed the approach to pediatric healthcare by instituting a free outpatient clinic within the hospital. His prominence rose during the tuberculosis epidemic, as he was the forerunner in detecting a cure using streptomycin. In the 1950s, Giovanni De Toni was acclaimed as a fervent supporter of breastfeeding. On 1 November 1965, the pediatrician left his role at the Italian pediatric institution. He subsequently became president of the Italian Society of Pediatrics (SIP), a position he held from 1966 to the time of his death in Genoa on 8 January 1973. This Special Issue is dedicated to his legacy at the Gaslini Children’s Hospital and to his descendants, Ettore and Teresa, who followed in his footsteps on a scientific journey.

Sixteen papers have been published in this Special Issue dedicated to his memory.

At the Università Cattolica del Sacro Cuore, Fondazione Policlinico “A. Gemelli”, Rome, Italy, Cera and colleagues addressed the hyperandrogenism in congenital adrenal hyperplasia (CAH) (46, XX simple-virilizing 21-OHD) [13]. CAH is a collection of autosomal recessive disorders that present with cortisol synthesis impairment. It results from a deficiency of one of the five enzymes needed for the synthesis of cortisol in the adrenal cortex [14,15,16].

Any medical intervention, particularly that involving a person’s gender identity, must, nevertheless, revolve around the patient’s needs, wants, and expectations, in addition to comprehensive facts.

Developmental abnormalities affecting the inner ear might lead to sensorineural hearing loss. In an REB-approved study on spontaneous abortions or tubal pregnancies conducted by researchers at the University of Split School of Medicine, Croatia, SOX2, JAGGED1, β-Catenin (CTNNB1), and Vitamin D Receptor (VDR) expression patterns were examined during the early development and innervation of the human inner ear [17]. SOX2 is a nuclear transcription factor with critical roles in stem cell and developmental biology [18,19]. They found that SOX2 is consistently more abundant than JAGGED1 in the human inner ear’s prosensory regions. At 6 weeks, vestibular prosensory domains were innervated, and at 10 weeks, this was extensive; finally, at 7–8 weeks, nerve fibers reached the base of the cochlear prosensory domain [17].

While related histological and molecular changes are poorly understood, tooth agenesis is thought to be caused by the failure of tooth germ-initiated tooth development [20,21,22]. In order to determine if tooth agenesis is linked to constitutively active FGF signaling, Wang et al. of Dalian Medical University, China, stimulated Fgf8 in dental mesenchyme using *Osr2*-*creKI* mice. Molar germs that were engineered with *Osr2-creKI* and *Rosa26R-Fgf8* showed elevated levels of Ectodin, Runx2, and Lef1, as well as reduced *Shh* transcription. The authors claimed that incisor agenesis is associated with ectopically activated *Fgf8* in dental mesenchyme, which in turn triggered incisor regression and postnatal molar microdontia [23].

A Shenzhen-based group from the Children’s Hospital, Institute of Advanced Technology, and the University of Chinese Academy of Sciences studied Osteocalcin (OCN) in chondrocyte differentiation and endochondral ossification on a CRISPR/Cas9 *bglap–bglap2*-deficient mouse model [24]. Yu and colleagues demonstrated the OCN’s regulatory involvement in chondrocyte development and endochondral ossification using a new CRISPR/Cas9-mediated *bglap-bglap2*-deficient (OCNem) animal model. During the neonatal and adolescent stages, the OCNem mice showed delayed primary and secondary ossification centers, longer cartilage in growth plates, and increased hypertrophic zones compared to wild-type rodents. These abnormalities showed that endochondral ossification was disrupted throughout embryonic and postnatal periods due to OCN deficiency [24].

With an aging population, the likelihood of various ailments is increasing, including sarcopenia, characterized by a steady decline in skeletal muscle mass, strength, and function [25,26,27,28,29]. A sarcopenia team group located primarily in Grenada, Spain, used zebrafish as an animal model [30]. They understood variations in muscle structure to be partly caused by a decline in the number of myocytes, and an increase in collagen with aging and changes in synthesis, degradation, and differentiation pathways. These changes were accompanied by mitochondrial modifications, including a decrease of nearly 50% in intermyofibrillar mitochondria, a 100% increase in mitochondrial damage, and a decrease in mitochondrial dynamics. This study provides the first empirical evidence that zebrafish can be used as a model to examine the age-related decline of skeletal muscle [30].

It is mostly unknown that *Acanthamoeba* trophozoites and *Toxocara canis* eggs can decrease tumors in both laboratory and animal settings. Despite long-standing knowledge of this fact, the exact process by which these parasites exert their anticancer effects remains a mystery. At the Hospital Infantil de México Federico Gómez (HIMFG), México City, Mexico, Maravelez Acosta and colleagues examined neuroblastoma (NB) SH-SY5Y cells using Western blotting, confocal microscopy, and immunofluorescence to determine whether or not they bind to antibodies against *Acanthamoeba* and *Toxocara canis* [31]. The authors found two 70 kDa fragments and one 60 kDa fragment using Western blotting, as well as two 115 kDa fragments and one 70 kDa fragment with the help of anti-*Toxocara canis* antibodies. Their research points to a possibility that these parasites and tumor cells share certain molecular similarities, which could aid in the removal of this kind of malignant tumor [31].

Takayasu’s arteritis (TAK) affects the aorta and its main branches via granulomatous inflammation, spreading insidiously and crippling patients. Although the exact cause of TAK is still unknown, what is known so far points to an autoimmune basis. A Beijing-based group highlighted the mounting evidence that B cells have far-reaching impacts on illness etiology and development than thought before [32]. Individuals with TAK have been found to undergo different changes in certain subsets of peripheral B cells. Recent decades have seen the success of B cell targeting treatment strategies, most notably rituximab, an anti-CD20 monoclonal antibody, in treating TAK [32].

It was only very recently that thrombotic microangiopathy (TMA) was linked to mutations in RNA exosome encoding. Wijnsma and colleagues, from Amalia Children’s Hospital, Radboud University Medical Center, Nijmegen, The Netherlands, report on a 4-month-old infant affected by Rotavirus-triggered TMA, harboring an *EXOSC5* gene mutation (c.230_232del p.Glu77del) [33]. The female newborn had the clinical pattern for CABAC syndrome, with cerebellar ataxia, brain abnormalities, and cardiac conduction problems. Four more infants who developed TMA following a viral trigger, and who had mutations in *EXOSC3*, were found in the literature. The authors conclude that TMA may occur even when complement dysregulation is not present if mutations in RNA exosome-influencing genes, such as *EXOSC3* and *EXOSC5*, cause neurodevelopmental and neurodegenerative diseases [33].

Research on the impact of fine dust on oral health is sparse, despite the fact that it is associated with a wide range of health problems, including cardiovascular, neurological, pulmonary, and malignant disorders [34,35,36]. In a Korean work, Kim and colleagues looked at the potential protective benefits on periodontal cells of exosomes created by mature hemp stem extract when exposed to fine dust [37]. By utilizing a range of techniques, such as microRNA profiling, PCR, flow cytometry, immunocytochemistry, enzyme-linked immunosorbent assay (ELISA), and Alizarin O staining, the authors discovered that periodontal cells regulated the expression of LL-37 and MCP-1, promoting the development of periodontal ligament stem cells into osteoblasts and other cells, and suppressing inflammatory genes while activating anti-inflammatory ones [37].

At the USC Keck School of Medicine, Los Angeles, CA, USA, Besaratinia and colleagues explored the utility of long non-coding RNAs (lncRNAs) for assessing the health consequences of vaping [38]. “Vaping”—the use of electronic cigarettes—is on the rise among young and adult smokers. However, the long-term effects of e-cigarette use on human health remain mostly unclear. The harmful chemicals in tobacco smoke and electronic cigarette vapor produce epigenetic alterations that can disrupt gene control, which in turn increases the risk of cancer and other diseases. There is a substantial amount of evidence suggesting that lncRNAs control genes are altered in smoking-related disorders. Moreover, the cells and tissues of vapers, and cells treated in vitro with e-cigarette aerosol extract, demonstrate deregulation of disease-related genes through lncRNAs, according to a small but increasing number of studies. In this review, the USA-based authors stress the importance of empirical evidence for tobacco product regulation and public health, identify the gaps in knowledge, and encourage future study [38].

Sepsis biomarkers were targeted in a narrative review by a group of Chinese universities [39]. Circular RNAs, HOXA distant transcript antisense RNA, microRNA-486-5p, protein C, triiodothyronine, and prokineticin 2 are some of the new biomarkers that have been found to be more sensitive and specific than C-reactive protein and procalcitonin, two of the most used traditional biomarkers in supportive care. These newly discovered indicators show great promise for the diagnosis and prognosis of sepsis in its early stages. The authors suggest the potential of customized treatment and the importance of multi-biomarker approaches in sepsis management [39].

A person’s capacity to adapt to hypoxic conditions is greatly associated with the occurrence of high-altitude disorders, such as acute mountain sickness (AMS), high-altitude cerebral edema (HACE), and high-altitude pulmonary edema (HAPE). In this Chinese study, Li and colleagues identified stanniocalcin 1 (*STC1*) as a critical gene impacting the onset of altitude sickness using functional studies and analysis of gene expression data [40]. The researchers discovered that there are significant changes in the expression of this gene across 13 different types of malignant tumors. These differences are linked to the hypoxic condition that exists inside the tumor microenvironment. STC1 also has a strong association with patient prognosis, and affects tumor immunity [40].

Although studies have shown that IL-16 single nucleotide polymorphisms (SNPs) affect the risk of many malignancies, no studies have examined their involvement in ovarian cancer. From the blood samples of 413 women of Central European heritage, comprising 200 patients with ovarian cancer and 213 healthy controls, four IL-16 SNPs were investigated using the PCR-RFLP (restriction fragment length polymorphism) method. An overwhelming majority of patients (62%), detected in late stages (FIGO IIb-IV, International Federation of Gynecology and Obstetrics), had high-grade serious ovarian cancer [41]. In this multinational study, Watroski and colleagues found that possible new genetic markers for increased OC risk in European women are rs11556218 genotypes that contain G [41]. Premenopausal women had the strongest associations with rs11556218 and rs4778889.

In vitro studies have demonstrated that arsenic trioxide (ATO) inhibits the growth of pancreatic neoplastic cells, while in vivo studies have demonstrated that ATO enhances the inhibitory effects of gemcitabine (Gem) on pancreatic cancer [42]. Unfortunately, ATO’s limited clinical use is due to its high toxicity, linked to the need for high dosages and indiscriminate targeting. An investigation carried out in Australia targeted the oncogenesis in vitro and in vivo (using a mouse model), conducted with this peptide-linked arsenic compound (PhAs-LHP), with a similar non-targeting arsenic chemical (phenylarsine oxide, PAO) and commercially available ATO [42]. This study provides more evidence that the combination of Gem and the peptide-linked arsenic chemical may be useful in the treatment of pancreatic cancer, as PhAs-LHP suppressed pancreatic cancer more effectively than ATO/PAO [42].

The pathophysiology of acute respiratory distress syndrome (ARDS) involves polymorphonuclear neutrophil granulocytes (PMN, leukocytes). In earlier research, it has been demonstrated that interaction with collagen III, a crucial component of lung tissue, is triggered by the generation of neutrophil reactive oxygen species (ROS) [43]. A German study assessed circulating PMNs (cPMNs) and tracheal secretion PMNs (tPMNs) from patients with and without ARDS with regard to function and phenotype to look for potential connections [43]. The authors used density-gradient gravity sedimentation to isolate cPMN, which required first endotracheal aspirate filtering. In patients with and without ARDS, the epitope distribution on cPMNs and tPMNs was considerably different. In general, tPMNs exhibited higher levels of CD66b, LOX-1, and fMLP-receptor expression, whereas cPMNs showed lower levels of CD62L expression [43]. Critical care physicians treating patients with ARDS can benefit greatly from this characterization of phenotypic and functional PMN.

Wilms tumor, the primary renal tumor detected in childhood, rarely occurs bilaterally and in dysplastic kidney [44,45,46,47]. A subtype of this kind of tumor, known as extrarenal teratoid Wilms’ tumor (TWT), has been described in fewer than 30 cases. Due to its complicated histology, it poses a substantial diagnostic problem and contains more than half heterologous tissue (in addition to the orthologous tissue). In a Canadian study, Alfawaz and colleagues detail a peculiar instance of a large mediastinal teratoma harboring nephroblastomatous components [48]. The pathology report showed nephroblastomatous components in addition to ectodermal, mesodermal, and endodermal tissues. Wilms Tumor 1 and other pertinent markers were detected by immunohistochemistry, which confirmed the diagnosis [48]. The anatomical and functional elements of nephrogenesis, as well as the variables that disturb it in embryonic kidneys, were highlighted in this paper’s in-depth examination of nephron formation (glomerulogenic zone) and nephrogenic resting, using the ratio between glomerulogenic zone and definitive zone [48].

This Special Issue, dedicated to the memory of Giovanni De Toni, presents sixteen innovative papers that reflect the pioneering spirit of this remarkable Italian pediatrician. Just as De Toni transformed pediatric healthcare through groundbreaking discoveries, like De Toni–Fanconi–Debré syndrome and innovative approaches to hospital management, these contemporary studies push the boundaries of pediatric medicine.

From molecular investigations into inner-ear development to novel therapeutic approaches for Takayasu’s arteritis, these contributions touch on both fundamental science and clinical applications. Tackling pressing modern challenges, such as the health implications of vaping and its environmental impacts on child health, they also explore promising new frontiers, including parasite-based tumor treatments and genetic insights into rare conditions.

These contributions collectively demonstrate how De Toni’s legacy continues to inspire progress in pediatric medicine. The diverse range of research, from bench to bedside, embodies his holistic approach to improving children’s healthcare through scientific innovation. As we face new medical challenges in the 21st century, this collection shows how the spirit of scientific inquiry and dedication to child health that De Toni exemplified remains vital in modern pediatric research.

Overall, the papers herein make excellent contributions to pediatrics and youth, cementing the memory of Professor G. De Toni for centuries to come. We are very grateful to the scientific community for participating in this initiative.

## References

[1] Liu D., Yu S., Zhang Y., Li Q., Kang P., Wang L., Han R., Cheng D., Chen A., Hou X. (2025). Fibroblast growth factor 23 predicts incident diabetic kidney disease: A 4.6-year prospective study. Diabetes Obes. Metab..

[2] Park M.Y., Tu C.L., Perie L., Verma N., Serdan T.D.A., Shamsi F., Shapses S., Heffron S., Gamallo-Lana B., Mar A.C. (2024). Targeted Deletion of Fibroblast Growth Factor 23 Rescues Metabolic Dysregulation of Diet-induced Obesity in Female Mice. Endocrinology.

[3] Fuchs M.A.A., Burke E.J., Latic N., Murray S.L., Li H., Sparks M.A., Abraham D., Zhang H., Rosenberg P., Saleem U. (2025). Fibroblast growth factor 23 and fibroblast growth factor receptor 4 promote cardiac metabolic remodeling in chronic kidney disease. Kidney Int..

[4] Wang Y., Zhang D., Zhou R., Yang X., Wang X., Jiang Y., Zhou X., Li D., Zhang J., Wu Y. (2025). Baseline fibroblast growth factor 23 predicts incident heart failure and cardiovascular mortality in patients with chronic kidney disease: A 3-year follow-up study. Int. J. Cardiol. Heart Vasc..

[5] Ning C., Kuhara T., Inoue Y., Zhang C.H., Matsumoto M., Shinka T., Furumoto T., Yokota K., Matsumoto I. (1996). Gas chromatographic-mass spectrometric metabolic profiling of patients with fatal infantile mitochondrial myopathy with de Toni-Fanconi-Debre syndrome. Acta Paediatr. Jpn..

[6] Ning C., Kuhara T., Matsumoto I. (1996). Simultaneous metabolic profile studies of three patients with fatal infantile mitochondrial myopathy-de Toni-Fanconi-Debre syndrome by GC/MS. Clin. Chim. Acta.

[7] Ogier H., Lombes A., Scholte H.R., Poll-The B.T., Fardeau M., Alcardi J., Vignes B., Niaudet P., Saudubray J.M. (1988). de Toni-Fanconi-Debre syndrome with Leigh syndrome revealing severe muscle cytochrome c oxidase deficiency. J. Pediatr..

[8] Van Biervliet J.P., Bruinvis L., Ketting D., De Bree P.K., Van der Heiden C., Wadman S.K. (1977). Hereditary mitochondrial myopathy with lactic acidemia, a De Toni-Fanconi-Debre syndrome, and a defective respiratory chain in voluntary striated muscles. Pediatr. Res..

[9] Hodrob T., Abusalameh A., Ismail I., Dweikat I., Rmeilah S.A., Sultan M., Libdeh B.A., Libdeh A.A., Shweiki S., Damseh N. (2025). Genetic, Clinical, and Biochemical Characterization of a Large Cohort of Palestinian Patients with Fanconi-Bickel Syndrome. Clin. Genet..

[10] Loon E., Lim N., Roldan G.A., Lake J. (2024). Tenofovir-associated Fanconi syndrome in liver transplant recipients with hepatitis B: A retrospective case series. Am. J. Transplant..

[11] Habuka M., Hosojima M., Yata Y., Kurumada K., Yamagiwa M., Yonezawa M., Sudo M., Kabasawa H., Ogawa A., Hama H. (2024). Fanconi syndrome with acute proximal tubular injury induced by a dietary supplement containing beni-koji: A case series report. BMC Nephrol..

[12] Farias F.H.G., Mhlanga-Mutangadura T., Guo J., Hansen L., Johnson G.S., Katz M.L. (2024). FAN1 Deletion Variant in Basenji Dogs with Fanconi Syndrome. Genes.

[13] Cera G., Corsello A., Novizio R., Di Donna V., Locantore P., Paragliola R.M. (2024). Severe Hyperandrogenism in 46,XX Congenital Adrenal Hyperplasia: Molecular Physiopathology, Late Diagnoses, and Personalized Management. Int. J. Mol. Sci..

[14] Panic Zaric S., Milenkovic T., Todorovic S., Mitrovic K., Cvetkovic D., Cehic M., Vekic J., Dumic K., Vukovic R. (2025). Metabolic Syndrome Spectrum in Children with Classic Congenital Adrenal Hyperplasia-A Comprehensive Review. Metabolites.

[15] Falhammar H., Lajic S., Nordenstrom A. (2025). Recent advances in treatments for congenital adrenal hyperplasia. Nat. Rev. Endocrinol..

[16] Anisowicz S.K., Vogt K.S. (2025). Congenital Adrenal Hyperplasia. Pediatr. Ann..

[17] Mikulić P., Ogorevc M., Petričević M., Kaličanin D., Tafra R., Saraga-Babić M., Mardešić S. (2024). SOX2, JAGGED1, β-Catenin, and Vitamin D Receptor Expression Patterns during Early Development and Innervation of the Human Inner Ear. Int. J. Mol. Sci..

[18] Hou Y., Nie Z., Jiang Q., Velychko S., Heising S., Bedzhov I., Wu G., Adachi K., Scholer H.R. (2025). Emerging cooperativity between Oct4 and Sox2 governs the pluripotency network in early mouse embryos. eLife.

[19] Abatti L.E., Gillespie Z.E., Lado-Fernandez P., Collado M., Mitchell J.A. (2025). A role for NFIB in SOX2 downregulation and epigenome accessibility changes due to long-term estrogen treatment of breast cancer epithelial cells. Biochem. Cell Biol..

[20] Borges G.H., Lins-Candeiro C.L., Henriques I.V., de Brito Junior R.B., Pithon M.M., Paranhos L.R. (2025). Exploring the genetics, mechanisms, and therapeutic innovations in non-syndromic tooth agenesis. Morphologie.

[21] Alamoudi R., Kanavakis G., Oeschger E.S., Halazonetis D., Gkantidis N. (2024). Occlusal characteristics in modern humans with tooth agenesis. Sci. Rep..

[22] Cammarata-Scalisi F., Willoughby C.E., El-Feghaly J.R., Tadich A.C., Castillo M.A., Alkhatib S., Elsherif M.A.E., El-Ghandour R.K., Coletta R., Morabito A. (2024). Main genetic entities associated with tooth agenesis. Clin. Oral. Investig..

[23] Wang Y., Wang J., Xu T., Yang S., Wang X., Zhu L., Li N., Liu B., Xiao J., Liu C. (2024). Ectopic Activation of Fgf8 in Dental Mesenchyme Causes Incisor Agenesis and Molar Microdontia. Int. J. Mol. Sci..

[24] Yu X.-F., Teng B., Li J.-F., Zhang J.V., Su Z., Ren P.-G. (2024). Novel Function of Osteocalcin in Chondrocyte Differentiation and Endochondral Ossification Revealed on a CRISPR/Cas9 bglap–bglap2 Deficiency Mouse Model. Int. J. Mol. Sci..

[25] Zhan Z., Zhang Y., Wu J., Lin J., Yan S. (2025). Predictive efficacy of different diagnostic criteria for sarcopenia in osteoporosis and fractures. Sci. Rep..

[26] Guimaraes L.T., da Silva M.Z.C., Reis N., Caramori J.C.T., Vogt B.P. (2025). Reliability between sarcopenia diagnosis by EWGSOP 1 and EWGSOP 2 criteria and association with clinical parameters in maintenance hemodialysis patients. J. Ren. Nutr..

[27] Lima A.B., Henrinques-Neto D., Scott D., de Araujo Pinto A., Dos Santos Ribeiro G., Peralta M., Miranda K.A., Campos P., Gouveia E.R. (2025). Relationship between physical function and sarcopenia in the older adults from Amazonas: A cross-sectional study. PLoS ONE.

[28] Liu Y., Guo Y., Xie S., Kong Y., Xu J. (2025). Association between bone mineral density T-score and respiratory sarcopenia in older adults. Front. Med..

[29] Chung J.Y., Kim S.G., Kim S.H., Park C.H. (2025). Sarcopenia: How to determine and manage. Knee Surg. Relat. Res..

[30] Aranda-Martínez P., Sayed R.K.A., Fernández-Martínez J., Ramírez-Casas Y., Yang Y., Escames G., Acuña-Castroviejo D. (2024). Zebrafish as a Human Muscle Model for Studying Age-Dependent Sarcopenia and Frailty. Int. J. Mol. Sci..

[31] Maravelez Acosta V.A., Garcia M.D., Patiño López G., Crisóstomo Vázquez M.D., Franco Sandoval L.O., Eligio García L. (2024). Association of Neuroblastoma (NB) SH-SY5Y Cells with Antibodies of Parasitic Origin (Anti-Acanthamoeba and Anti-Toxocara canis). Int. J. Mol. Sci..

[32] Guo S., Tian Y., Li J., Zeng X. (2024). A Glimpse into Humoral Response and Related Therapeutic Approaches of Takayasu’s Arteritis. Int. J. Mol. Sci..

[33] Wijnsma K.L., Schijvens A.M., Bouwmeester R.N., Aarts L.A.M., van den Heuvel L.P., Haaxma C.A., van de Kar N.C.A.J. (2024). Mutations in Genes Encoding Subunits of the RNA Exosome as a Potential Novel Cause of Thrombotic Microangiopathy. Int. J. Mol. Sci..

[34] Gupta M., Agrawal R.P., Meena B.L., Ramesh, Meel J.K., Agrawal R. (2022). Association between Long Term Exposure to Air Pollution, Impaired Fasting Glucose, Impaired Glucose Tolerance and Prevalence of Diabetes. J. Assoc. Physicians India.

[35] Ettler V., Stepanek D., Mihaljevic M., Drahota P., Jedlicka R., Kribek B., Vanek A., Penizek V., Sracek O., Nyambe I. (2020). Slag dusts from Kabwe (Zambia): Contaminant mineralogy and oral bioaccessibility. Chemosphere.

[36] Rupf S., Berger H., Buchter A., Harth V., Ong M.F., Hannig M. (2015). Exposure of patient and dental staff to fine and ultrafine particles from scanning spray. Clin. Oral. Investig..

[37] Kim E., Park Y., Yun M., Kim B. (2024). Functions of Hemp-Induced Exosomes against Periodontal Deterioration Caused by Fine Dust. Int. J. Mol. Sci..

[38] Besaratinia A., Blumenfeld H., Tommasi S. (2024). Exploring the Utility of Long Non-Coding RNAs for Assessing the Health Consequences of Vaping. Int. J. Mol. Sci..

[39] He R.-R., Yue G.-L., Dong M.-L., Wang J.-Q., Cheng C. (2024). Sepsis Biomarkers: Advancements and Clinical Applications—A Narrative Review. Int. J. Mol. Sci..

[40] Li Q., Xu Z., Gong Q., Shen X. (2024). Identification and Validation of STC1 Act as a Biomarker for High-Altitude Diseases and Its Pan-Cancer Analysis. Int. J. Mol. Sci..

[41] Watrowski R., Schuster E., Van Gorp T., Hofstetter G., Fischer M.B., Mahner S., Polterauer S., Zeillinger R., Obermayr E. (2024). Association of the Single Nucleotide Polymorphisms rs11556218, rs4778889, rs4072111, and rs1131445 of the Interleukin-16 Gene with Ovarian Cancer. Int. J. Mol. Sci..

[42] He H., Dumesny C., Carrall J.A., Dillon C.T., de Roo K.I., Eutick M., Dong L., Baldwin G.S., Nikfarjam M. (2024). A Tumor Homing Peptide-Linked Arsenic Compound Inhibits Pancreatic Cancer Growth and Enhances the Inhibitory Effect of Gemcitabine. Int. J. Mol. Sci..

[43] Kraus R.F., Ott L., Utpatel K., Kees M.G., Gruber M.A., Bitzinger D. (2024). Neutrophils in the Spotlight—An Analysis of Neutrophil Function and Phenotype in ARDS. Int. J. Mol. Sci..

[44] Perotti D., O’Sullivan M.J., Walz A.L., Davick J., Al-Saadi R., Benedetti D.J., Brzezinski J., Ciceri S., Cost N.G., Dome J.S. (2025). Hallmark discoveries in the biology of non-Wilms tumour childhood kidney cancers. Nat. Rev. Urol..

[45] Somers G.R., L’Hermine-Coulomb A., Matoso A., O’Sullivan M.J. (2025). Paediatric renal tumours: An update on challenges and recent developments. Virchows Arch..

[46] Sergi C., Kos M. (2018). Bilateral Wilms’Tumor in Trisomy 18 Syndrome: Case Report and Critical Review of the Literature. Ann. Clin. Lab. Sci..

[47] Oddone M., Marino C., Sergi C., Occhi M., Negri F., Kotitza Z., De Bernardi B., Jasonni V., Toma P. (1994). Wilms’ tumor arising in a multicystic kidney. Pediatr. Radiol..

[48] Alfawaz B., Koujok K., Eamer G., Sergi C.M. (2024). Mediastinal Teratoma with Nephroblastomatous Elements: Case Report, Literature Review, and Comparison with Maturing Fetal Glomerulogenic Zone/Definitive Zone Ratio and Nephrogenic Rests. Int. J. Mol. Sci..

